# The Effect of Maternal Race, Ethnicity, and Nativity on Macrosomia Among Infants Born in the United States

**DOI:** 10.7759/cureus.39391

**Published:** 2023-05-23

**Authors:** Janardhan Mydam, Pranav Mellacheruvu, Brahm Coler, Soumini Chintala, Kiran S Depala, Shreeya Sangani

**Affiliations:** 1 Department of Neonatology, John H. Stroger, Jr. Hospital of Cook County, Chicago, USA; 2 Elson S. Floyd College of Medicine, Washington State University, Spokane, USA; 3 Department of Pediatrics, Phoenix Children's Hospital, Phoenix, USA; 4 Department of Public Health, Saint Louis University, St Louis, USA

**Keywords:** neonatalogy, infants, nativity, ethnicity, race, macrosomia

## Abstract

Objectives: This study aims to elucidate the influence of race, ethnicity, and nativity on macrosomia rates, hypothesizing that higher rates are observed among White non-Latina mothers and United States (US)-born mothers.

Study Design: We analyzed data from 1,791,718 US births sourced from the National Center for Health Statistics of the Centers for Disease Control and Prevention. Logistic regression analyses were conducted to examine the associations between macrosomia rates and maternal race, ethnicity, and nativity.

Results: After excluding non-singleton, preterm, post-term births, and those with missing data, six maternal cohorts were identified: White non-Latina US-born (1,147,096) and foreign-born (75,542), Black non-Latina US-born (174,540) and foreign-born (32,200), and Latina US-born (223,968) and foreign-born (137,515). White non-Latina US-born mothers had the highest rates of excessive gestational weight gain (58.9%). Black non-Latina US-born mothers exhibited the highest rates of pre-pregnancy diabetes (0.7%) and obesity (29.5%). Macrosomia rates were highest among White non-Latina US-born mothers (10.7%) compared to other cohorts. After adjusting for socioeconomic and health-related factors, this group maintained the highest odds of macrosomia (OR: 1.876; 95%CI 1.832-1.922, P<0.001).

Conclusion: Our findings reveal that White non-Latina US-born mothers experience the highest macrosomia rates, which persist after adjusting for known confounders. These results have significant implications for the development of gestational surveillance tools and targeted public health interventions aimed at improving pregnancy outcomes among high-risk cohorts.

## Introduction

Fetal macrosomia is defined as a birth weight greater than 4000g or greater than the 90th percentile for gestational age [1,2]. According to the most recent birth data published by the Centers for Disease Control and Prevention (CDC), approximately 7% of infants born in the United States (US) in 2017 had a birth weight >4,000g, while around 1% had a birth weight >4,500g [3]. Birth weights >5,000g were exceedingly rare (approximately 0.1%) [3]. Globally, rates of macrosomia are rising in tandem with increasing rates of diabetes and obesity [4]. Macrosomia is associated with adverse pregnancy outcomes for both the mother and infant, with adverse outcomes increasing proportionally to the degree of macrosomia [1]. Mothers face a higher risk of emergency Caesarean section, obstetrical trauma, and postpartum hemorrhage, while macrosomic neonates may experience birth trauma, shoulder dystocia, brachial plexus and skeletal injuries, asphyxia, and higher morbidity and mortality rates than neonates with lower birth weights [1,5,6]. Furthermore, large-for-gestational-age (LGA) infants, both male and female, are at higher risk for obesity in later life, with an increased propensity for giving birth to an LGA infant themselves [7].

The mechanism of fetal overgrowth involves the transplacental passage of glucose and subsequent stimulation of insulin secretion by the fetal pancreas. The combination of hyperglycemia and hyperinsulinemia produces an increase in fetal protein and fat stores, resulting in macrosomia [8,9]. It has been well established that underlying maternal metabolic disorders, gestational weight gain, and diabetes have the greatest impact on the development of LGA infants [10-12]. The risk of macrosomia increases in proportion to BMI, with mothers at BMIs ≥ 30 having the highest risk of delivering an LGA infant [7,11,13]. Notably, mothers who have excessive gestational weight gain are up to 3.5 times more likely to have a macrosomic newborn [14]. Among normal-weight and underweight mothers, pre-gestational diabetes is thought to be the greatest contributor to elevated macrosomia risk [11,13]. Babies born to diabetic mothers have a three-fold higher rate of developing macrosomia compared to normoglycemic mothers [9].

Other factors that influence macrosomia risk include older maternal age, male gender of infant, post-term pregnancy, and a previous macrosomic infant [1,5,6,15-17]. While these clinical risk factors are well-established, the sociodemographic determinants of macrosomia remain inadequately studied and are far less defined [18]. Furthermore, it remains to be seen whether maternal sociodemographic differences influence macrosomia risk independent of these risk factors. Currently, there is not a widely recognized or verified combination of clinical and social/demographic factors that can accurately predict those infants who are at greatest risk for macrosomia and its subsequent morbidities. Since greater maternal height and weight correlate with a higher infant birth weight, it has been posited that demographic factors that influence maternal BMI may also influence the risk of macrosomia; accordingly, maternal country of birth, genetics, race, and ethnicity each impact human height and weight, prompting interest in their potential impact on macrosomia risk. Moreover, the role of various demographic variables (e.g., maternal smoking status and maternal age, as well as maternal race, ethnicity, and nativity) have been studied extensively in neonates born prematurely or with low birth weight (LBW) [18,19]. Our previous study aimed to enrich the existing literature by evaluating the incidence of LBW infants in the US stratified by maternal race, ethnicity, and nativity. We emphasized the understudied aspect of Latina mothers stratified by race, thus addressing a critical gap in the current research [20]. In this current study, we aimed to assess the incidence of macrosomia among infants born to mothers in the US, stratified by race, ethnicity, and nativity.

This study evaluated the hypotheses that the incidence of macrosomia is higher among (a) White mothers compared to other races, (b) non-Latina mothers compared to Latina mothers, and (c) mothers born in the US compared to mothers born outside of the US. The primary objective was to calculate the incidence of macrosomia among cohorts of mothers stratified by race, ethnicity, and nativity, while the secondary objective was to calculate the odds ratios (ORs) for developing macrosomia in these different groups after adjusting for known clinical risk factors.

## Materials and methods

Data and study population

Natality data for 11,862,780 live births among infants born in the US between 2011 and 2013 were obtained from the National Center for Health Statistics (NCHS) of the CDC. The de-identified data were accessed in accordance with CDC data use agreements through a special request process. As the provided dataset did not include patient identifiers, this study received Institutional Review Board (IRB) exemption.

The extracted data encompassed various factors, such as birth weight, sex of the infant, gestational age at birth, and maternal characteristics including age, race, ethnicity, nativity, and socioeconomic factors (e.g., education status, gestational weight gain, obesity, smoking history, specific pregnancy-related disorders or complications such as diabetes and hypertension, and the timeline of prenatal care). Exclusions were applied to non-singleton births, singleton preterm births, births with unstated gestational age, and births from the 14 US states that either did not collect data for one or more study variables or collected data in a non-standard format. The following US states were excluded from the study due to missing data: Alabama, Arkansas, Alaska, Arizona, Connecticut, Hawaii, Maine, New Jersey, Rhode Island, West Virginia, Michigan, Mississippi, Georgia, and Virginia. 

**Figure 1:**
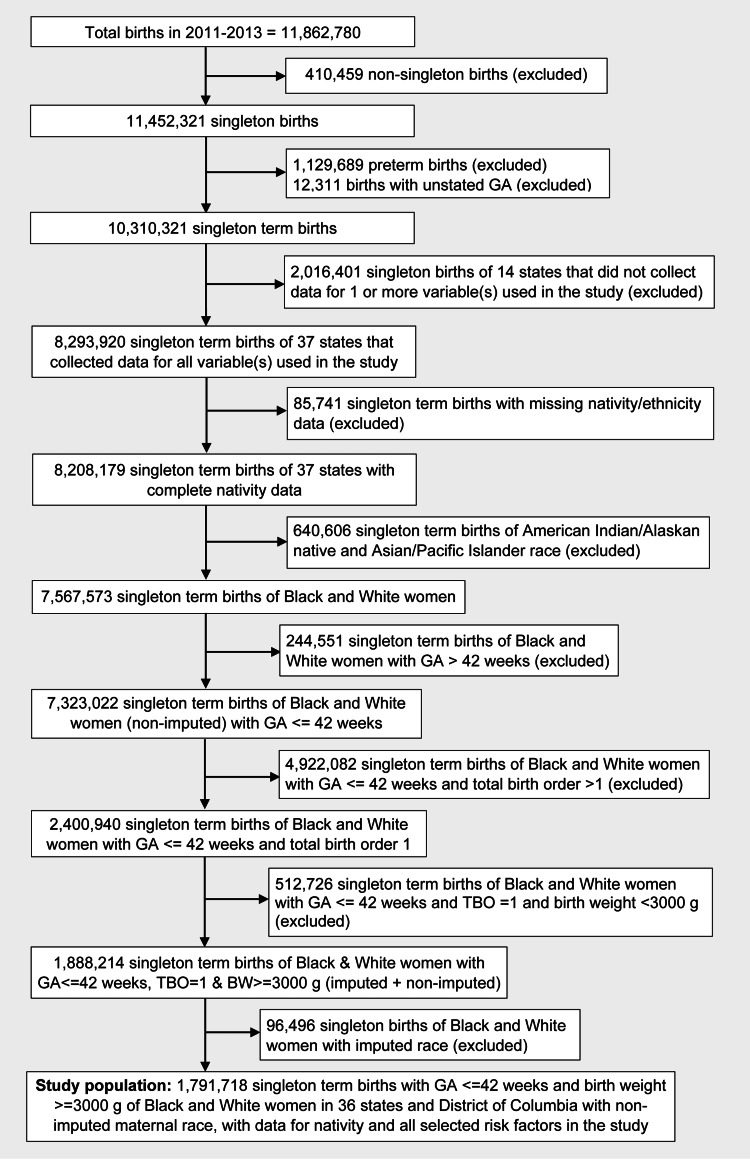
Flowchart for selection of study population from the 2011–2013 National Centers for Health Statistics (NCHS) natality database. BW, birth weight; GA, gestational age; TBO, total birth order.

Infants with missing nativity data or mothers with any self-identified race other than Black or White (e.g., Asian/Pacific Islander, or Indian/Alaskan Native) were excluded, as there are currently insufficient birth and census data on these populations, some of which comprise significantly smaller portions of the US population. Additionally, singleton term births of Black and White mothers with any of the following criteria were excluded: a gestational age >42 weeks; a gestational age ≤42 weeks and a total birth order >1; a gestational age ≤42 weeks, a total birth order >1, and a birth weight <3000g; or a gestational age ≤42 weeks, a total birth order >1, and a birth weight <3000g where maternal race was imputed using statistical methods (Figure 1). Mothers with imputed race were excluded because the aim was to investigate birth outcomes of mothers who clearly self-identified their race and ethnicity as White non-Latina or Black non-Latina.

Study variables and group classification

The primary outcome variable in this study was birth weight, specifically focusing on whether the infant had fetal macrosomia (defined as a birth weight ≥4000g). To enable comparison with the current literature and due to insufficient birth and census data among certain racial populations, data analysis was limited to mothers who self-identified as Black or White, as recorded on the infant's birth certificate. Regarding ethnicity identification (i.e., Latina or non-Latina), infants of Latina US-born and Latina foreign-born mothers were not further stratified by race (i.e., Black or White) to maintain consistency and comparability with similar studies utilizing natality data. Maternal nativity variables were foreign-born and US-born, with the latter defined as birth within any of the 50 states (including New York City and the District of Columbia).

The independent (or "group") variable of interest for analysis was derived from mothers representing six different cohorts of maternal race, ethnicity, and nativity: White US- and foreign-born non-Latina individuals, Black US- and foreign-born non-Latina individuals, and Latina US-born and Latina foreign-born.

Other independent variables included marital status, paternal acknowledgment, and maternal characteristics such as age (categorized as ≤19, 20-24, 25-29, 30-34, and ≥35 years), education (<12 years or ≥12 years), gestational weight gain (classified as appropriate, below appropriate, or above appropriate, as defined by the American College of Obstetrics and Gynecologists), gestational age (37-40 weeks or >40 weeks), and obesity (yes (BMI≥30) or no (BMI≤30)) [21]. The timing of prenatal care initiation was assessed using the trimester of entry into care, and the evaluation of medical disease during pregnancy was based on the presence or absence of gestational diabetes, pre-gestational diabetes, gestational hypertension, pre-pregnancy hypertension, and eclampsia. A sub-group analysis was performed to assess the relationship between macrosomia rates and maternal pre-gestational diabetes or gestational diabetes. Maternal tobacco use and the use of special supplemental nutrition for women, infants, and children (WIC) were also evaluated.

Only 857 (0.05%) births had missing data for birth weight. Among the 14 risk factors studied, the two variables with the highest percentages of missing values were gestational weight gain (4.43%) and obesity (3.03%), while the variables of race, nativity, maternal age, marital status, sex of infant, and gestational age had no missing values (Table 1).

**Table 1:** Percentage of analyzed and missing observations for selected variables in the study population, United States, 2011–2013. Singleton term births with a gestational age ≤42 weeks and a birth weight ≥3000g to black or white mothers from 36 states of the United States (plus the District of Columbia) who had non-imputed race and data for nativity and all selected risk factors WIC, special supplemental nutrition for women, infants, and children

Variable	Observations	
	Analyzed %	Missing %
Low birth weight	99.95	0.05
Race and nativity	100.00	0.00
Maternal age	100.00	0.00
Maternal education	99.45	0.55
Marital status	100.00	0.00
First trimester initiation of prenatal care	97.47	2.53
Paternal acknowledgement	99.71	0.29
WIC recipient	98.44	1.56
Gender of infant	100.00	0.00
Medical disease during pregnancy	99.58	0.42
Smoking cigarettes	99.36	0.64
Weight gain during pregnancy	95.57	4.43
Pre-pregnancy diabetes	99.58	0.42
Gestational diabetes	99.58	0.42
Gestational age	100.00	0.00
Obesity	96.97	3.03

Statistical analysis

The statistical correlation between maternal characteristics (e.g., maternal race, ethnicity, and nativity) and the incidence of fetal macrosomia was analyzed using chi-square (χ²) tests. We investigated the association between each group (identified by maternal race, ethnicity, and nativity) and fetal macrosomia using four different logistic regression models in which Black non-Latina US-born mothers served as the reference group. Model 1 was a crude analysis that included only the "group" variable (unadjusted model), while Model 2 adjusted for the effects of measured demographic and medical risk factors for fetal macrosomia (including obesity, gestational weight gain, pre-pregnancy diabetes, gestational diabetes, and maternal age). Model 3 added major socioeconomic risk factors for fetal macrosomia (including maternal education, marital status, paternal acknowledgment, and WIC status) to Model 2. Model 4 adjusted for all factors in Model 3 and for prenatal care initiation, medical disease during pregnancy, sex of infant, and gestational age. Finally, Model 5 adjusted for maternal smoking status. All data processing/preparation and statistical analyses were conducted using SAS version 9.4 (2013; SAS Institute Inc. Cary, North Carolina, United States).

## Results

Study population and data availability

After applying numerous exclusion criteria detailed in Figure 1, including maternal data from the NCHS natality database that featured imputed race rather than self-identified race (Table 2), the original total sample population of 11,862,780 was reduced to 1,791,718 births for analysis.

The 1,791,718 mothers analyzed in this study were categorized into six groups based on race, ethnicity, and nativity: White non-Latina US- and foreign-born mothers (1,147,096 and 75,542, respectively), Black non-Latina US- and foreign-born mothers (174,540 and 32,200, respectively), and Latina US-born and Latina foreign-born mothers (223,968 and 137,515, respectively).

**Table 2:** Distribution (%) of non-imputed and imputed race among Latina and non-Latina mothers and Latina mothers subdivided by race and nativity in unrestricted and restricted NCHS singleton natality data, United States, 2011–2013. a. Unrestricted data contains 2011–2013 NCHS natality data for singleton live births of US-born and foreign-born mothers (excluding unknown/missing nativity) for all 50 states (including 14 states that did not collect data for one or more variable(s) used in the study) plus District of Columbia. b. Restricted data contains 2011–2013 NCHS natality data for singleton live births of US-born and foreign-born mothers (excluding unknown/missing nativity) for 36 states (excluding 14 states that did not collect data for one or more variable(s) used in the study) plus District of Columbia. ** p-value < 0.01; *** p-value < 0.001 NCHS, National Center for Health Statistics; US, United States

Study groups by race, ethnicity, and nativity	Model 1^a^ n = 1,790,861		Model 2^b^ n = 1,709,836		Model 3^c^ n = 1,680,004		Model 4^d^ n = 1,647,405			Model 5^e^ n = 1,651,406	
	OR	95% CI	OR	95% CI	OR	95% CI	OR	95% CI	OR		95% CI
Black non-Latina US-born	1.00		1.00		1.00		1.00			1.00	
Latina Foreign-born	1.374***	(1.336,1.414)	1.535***	(1.490,1.581)	1.536***	(1.490,1.584)	1.527***	(1.480,1.575)		1.531***	(1.484,1.579)
Latina US-born	1.362***	(1.328,1.398)	1.437***	(1.399,1.476)	1.406***	(1.368,1.444)	1.407***	(1.368,1.446)		1.403***	(1.365,1.442)
Black non-Latina Foreign-born	1.532***	(1.465,1.601)	1.582***	(1.509,1.658)	1.565***	(1.492,1.641)	1.559***	(1.475,1.627)		1.550***	(1.486,1.638)
White non-Latina Foreign-born	1.781***	(1.726,1.838)	1.829***	(1.769,1.892)	1.739***	(1.681,1.800)	1.765***	(1.704,1.828)		1.709***	(1.650,1.770)
White non-Latina US-born	1.993***	(1.951,2.035)	1.911***	(1.869,1.955)	1.819***	(1.777,1.861)	1.876***	(1.832,1.922)		1.788***	(1.747,1.831)

Risk factors for fetal macrosomia

Table 3 displays the prevalence of risk factors for macrosomia among racial (Black or White) and ethnic (Latina or non-Latina) cohorts of mothers according to the nativity. Several risk factors were more frequent among White non-Latina foreign-born mothers compared to White non-Latina US-born, Black non-Latina US-born, and Latina US-born mothers. A higher percentage of White foreign-born mothers were aged ≥35 years (15.6%), had ≥12 years of education (95.5%), were married (84.0%) with a paternal acknowledgment (84.1%), and did not receive WIC (75.2%). White US-born mothers had the highest rates of above-appropriate gestational weight gain (58.9%) and first-trimester initiation of prenatal care (81.4%). Black US-born mothers exhibited the highest rates of pre-pregnancy diabetes (0.7%) and obesity (29.5%). Black foreign-born mothers had the highest rate of gestational diabetes (4.8%). In contrast, the risk profile was generally lower among Latina mothers (both US- and foreign-born). The proportions of mothers with no medical diseases, no cigarette smoking, and infant gestational age of >40 weeks were roughly similar among mothers of all races and ethnicities (Table 3).

**Table 3:** Distribution (%) of selected risk factors among Black, White, and Latina mothers according to nativity, United States, 2011–2013 FB, foreign-born; PNC, prenatal care; USB, US-born; WIC, special supplemental nutrition for women, infants, and children. *Indicates high-risk categories for fetal macrosomia

Maternal Characteristics	Black		White		Latina	
	USB %	FB %	USB %	FB %	USB %	FB %
Maternal age						
<=19 years	31.13	5.42	12.12	3.13	32.85	18.30
20-24 years	41.50	22.20	27.55	18.57	36.94	32.58
25-29 years	16.35	34.84	31.96	32.76	17.74	25.98
30-34 years	7.77	25.57	21.28	29.93	9.23	15.38
≥35 years*	3.26	11.96	7.09	15.62	3.24	7.77
Maternal education						
<12 years	21.25	12.10	8.14	4.46	22.86	32.72
≥12 years*	78.75	87.90	91.86	95.54	77.14	67.28
Marital status						
Yes*	16.00	56.09	64.25	84.03	32.92	45.68
No	84.00	43.91	35.75	15.97	67.08	54.32
First trimester PNC						
Yes*	66.12	60.42	81.39	77.38	72.95	69.36
No	33.88	39.58	18.61	22.62	27.05	30.64
Paternal acknowledgement						
Yes (married)*	16.11	56.33	64.43	84.14	32.97	45.73
Yes (unmarried)	46.97	27.11	26.26	12.18	50.86	40.76
No	36.92	16.56	9.30	3.68	16.17	13.51
WIC						
Yes	73.27	58.99	30.63	24.82	67.61	68.91
No*	26.73	41.01	69.37	75.18	32.39	31.09
Sex of infant						
Female	45.62	45.74	46.83	46.55	47.04	46.89
Male*	54.38	54.26	53.17	53.45	52.96	53.11
Medical diseases						
Yes	7.50	4.61	6.59	3.15	4.06	3.44
No*	92.50	95.39	93.41	96.85	95.94	96.56
Cigarette smoking						
Yes	4.10	0.38	8.94	2.00	2.07	0.32
No*	95.90	99.62	91.06	98.00	97.93	99.68
Weight gain during pregnancy						
Appropriate	24.76	30.57	28.47	34.06	28.40	34.09
Below appropriate	16.50	22.68	12.59	16.28	15.38	20.11
Above appropriate*	58.74	46.75	58.94	49.66	56.22	45.80
Pre-pregnancy diabetes						
No	99.34	99.52	99.50	99.69	99.57	99.60
Yes*	0.66	0.48	0.50	0.31	0.43	0.40
Gestational diabetes						
No	96.78	95.22	96.16	95.97	97.03	96.26
Yes*	3.22	4.78	3.84	4.03	2.97	3.74
Gestational age						
37–40 weeks	83.85	81.62	79.89	80.30	83.07	82.29
> 40 weeks*	16.15	18.38	20.11	19.70	16.93	17.71
Obesity (BMI ≥ 30)						
Yes*	29.49	16.25	19.53	8.79	23.26	14.59
No	70.51	83.75	80.47	91.21	76.74	85.41

Incidence of fetal macrosomia

**Figure 2:**
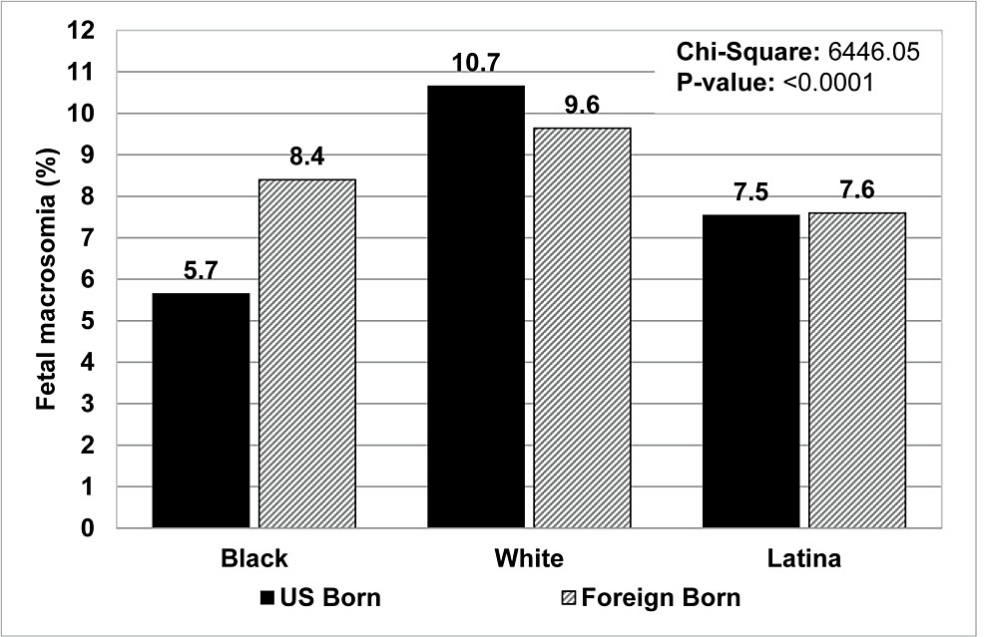
Distribution of fetal macrosomia rates among Black, White, and Latina mothers according to maternal nativity, United States, 2011-2013.

When stratified by ethnicity and nativity, the incidence of fetal macrosomia was 10% for US-born non-Latina mothers and 9.27% for foreign-born non-Latina mothers. There was no significant difference in fetal macrosomia rates between US-born and foreign-born Latina mothers (7.54% and 7.6%, respectively) (Table 4). When non-Latina mothers were stratified by ethnicity, nativity, and race, White US-born non-Latina mothers had the highest incidence of fetal macrosomia (10.66%) followed closely by White foreign-born non-Latina mothers (9.64%). Thus, Latina mothers (of combined Black and White races) who were either US- or foreign-born had lower rates of fetal macrosomia compared with White non-Latinx mothers but were found to have higher rates of macrosomia compared to Black non-Latina US-born mothers (5.65%) who had the lowest rate among all stratified cohorts. Black non-Latina foreign-born mothers had a markedly higher incidence of macrosomia (8.4%) than Black non-Latina US-born mothers and were more similar to the rates seen with Latina foreign-born mothers (8.1%) (Table 4, Figure 2). Mothers with pregestational diabetes or gestational diabetes were assessed from each racial, ethnic, and nativity cohort; distribution rates of macrosomia among these groups remained similar compared to those without a history of pregestational diabetes or gestational diabetes (Table 5). Macrosomia rates among the excluded racial or ethnic cohorts (e.g., non-Latina American Indian or Alaskan Native, Latina American Indian or Alaskan Native, non-Latina Asian or Pacific Islander, and Latina Asian or Pacific Islander) have been included in Table 6.

**Table 4:** Distribution of fetal macrosomia rates among mothers stratified by maternal (i) ethnicity and nativity, and (ii) ethnicity, nativity and race, United States, 2011–2013. *Defined as a birth weight ≥4000 g

	Fetal macrosomia* ≥4000 g)	
	Yes %	No %
Stratified by maternal ethnicity and nativity		
Latina US-born	7.54	92.46
Latina foreign-born	7.60	92.40
Non-Latina US-born	10.00	90.00
Non-Latina foreign-born	9.27	90.73
Stratified by maternal ethnicity, nativity and race		
Latina US-born	7.22	92.78
Latina foreign-born	8.10	91.90
White Non-Latina US-born	10.66	89.34
White Non-Latina foreign-born	9.64	90.36
Black Non-Latina US-born	5.65	94.35
Black Non-Latina foreign-born	8.40	91.60

**Table 5:** Macrosomia rates among different groups of diabetic mothers by race, ethnicity, and nativity US: United States

	Macrosomia	
	Yes	No
Pre-pregnancy diabetes		
Non-Latina White US Born	1328 (23.10)	4420 (76.90)
Non-Latina White Foreign Born	42 (18.26)	188 (81.74)
Latina US Born	192 (19.88)	774 (80.12)
Latina Foreign Born	82 (15.10)	461 (84.90)
Non-Latina Black US Born	199 (17.24)	955 (82.76)
Non-Latina Black Foreign Born	25 (16.23)	129 (83.77)
Gestational diabetes		
Non-Latina White US Born	5554 (12.67)	38299 (87.33)
Non-Latina White Foreign Born	333 (10.98)	2700 (89.02)
Latina US Born	812 (12.22)	5832 (87.78)
Latina Foreign Born	566 (11.01)	4576 (88.99)
Non-Latina Black US Born	684 (12.19)	4926 (87.81)
Non-Latina Black Foreign Born	227 (14.85)	1302 (85.15)

**Table 6:** Fetal macrosomia rates among mothers stratified by maternal (i) race (ii) ethnicity and race, and (iii) ethnicity, race, and nativity, expanded to incorporate additional maternal races, United States, 2011–2013.

	Macrosomia	
	Yes	No
Maternal Race		
Black	156100 (9.92)	1417151 (90.08)
White	13375 (6.15)	204235 (93.85)
American Indian / Alaskan Native	2111 (11.15)	16821 (88.85)
Asian / Pacific Islander	8135 (5.62)	136627 (94.38)
Maternal Ethnicity and Race		
Non- Latina White	129562 (10.60)	1093076 (89.40)
Latina White	26538 (7.57)	324075 (92.43)
Non- Latina Black	12566 (6.08)	194174 (93.92)
Latina Black	809 (7.44)	10061 (92.56)
Non- Latina American Indian/Alaskan Native	1901 (11.53)	14589 (88.47)
Latina American Indian/Alaskan Native	210 (8.60)	2232 (91.40)
Latina Asian/Pacific Islander	7984 (5.61)	134399 (94.39)
Non- Latina Asian/Pacific Islander	151 (6.35)	2228 (93.65)
Maternal Ethnicity Race and Nativity		
Non- Latina White US Born	122282 (10.66)	1024814 (89.34)
Non- Latina White Foreign Born	7280 (9.64)	68262 (90.36)
Latina Whites US Born	16386 (7.56)	200252 (92.44)
Latina Whites Foreign Born	10152 (7.58)	123823 (92.42)
Non- Latina Black US Born	9861 (5.65)	164679 (94.35)
Non- Latina Black Foreign Born	2705 (8.40)	29495 (91.60)
Latina Black US Born	504 (6.88)	6826 (93.12)
Latina Black Foreign Born	305 (8.62)	3235 (91.38)
Non- Latina American Indian/Alaskan Native US Born	1875 (11.52)	14406 (88.48)
Non- Latina American Indian/Alaskan Native Foreign Born	26 (12.44)	183 (87.56)
Latina American Indian/Alaskan Native US Born	192 (8.85)	1978 (91.15)
Latina American Indian/Alaskan Native Foreign Born	18 (6.62)	254 (93.38)
Non- Latina Asian/Pacific Islander US Born	1637 (5.80)	26611 (94.20)
Non- Latina Asian/Pacific Islander Foreign Born	6347 (5.56)	107788 (94.44)
Latina Asian/Pacific Islander US Born	111 (6.41)	1621 (93.59)
Latina Asian/Pacific Islander Foreign Born	40 (6.18)	607 (93.82)

Logistic regression analysis, using Black non-Latina US-born mothers as the reference group, showed that White non-Latina US- and foreign-born, Black non-Latina foreign-born, and Latina US- and foreign-born mothers had higher odds of delivering a macrosomic baby than the Black non-Latina US-born (P<0.001), regardless of the model used in the analysis (Table 7). For each maternal group studied (except White non-Latina US-born), the ORs for fetal macrosomia increased slightly after adjusting for maternal demographic and social covariates (Model 2). The ORs decreased slightly after constructing Model 3 (adjusting for socioeconomic risk factors in addition to Model 2), Model 4 (which adjusted for first-trimester initiation of prenatal care, medical disease during pregnancy, sex of infant, and gestational age in addition to Model 3), and Model 5 (which further adjusted for maternal smoking status in addition to Model 4).

**Table 7:** Odds ratios for the effect of maternal race and nativity on macrosomia among Black, White, and Latina mothers, adjusting for different sets of risk factors in multivariable logistic regression models with Black non-Latina US-born mothers as the reference group, United States, 2011–2013. a. Model 1 was a crude analysis (unadjusted model), included only the Group variable (the Group variable represents the 6 study groups of race and nativity categories). b. Model 2 adjusted for obesity, weight gain during pregnancy, pre-pregnancy diabetes, gestational diabetes and maternal age. c. Model 3 adjusted for Model 2 covariates plus socio-economic factors (maternal education, marital status, paternal acknowledgment, and WIC recipient). d. Model 4 adjusted for Model 3 covariates plus first trimester initiation of prenatal care, medical disease during pregnancy, sex of infant, and gestational age. e. Model 5 adjusted for Model 4 covariates plus maternal smoking status. ** p-value < 0.01, *** p-value < 0.001 CI, confidence interval; OR, odds ratio; WIC, special supplemental nutrition for women, infants, and children, US: United States

Study groups by race, ethnicity, and nativity	Model 1^a^ n = 1,790,861		Model 2^b^ n = 1,709,836		Model 3^c^ n = 1,680,004		Model 4^d ^ n = 1,647,405			Model 5^e ^ n = 1,651,406	
	OR	95% CI	OR	95% CI	OR	95% CI	OR	95% CI	OR		95% CI
Black non-Latina US-born	1.00		1.00		1.00		1.00			1.00	
Latina Foreign-born	1.374***	(1.336,1.414)	1.535***	(1.490,1.581)	1.536***	(1.490,1.584)	1.527***	(1.480,1.575)		1.531***	(1.484,1.579)
Latina US-born	1.362***	(1.328,1.398)	1.437***	(1.399,1.476)	1.406***	(1.368,1.444)	1.407***	(1.368,1.446)		1.403***	(1.365,1.442)
Black non-Latina Foreign-born	1.532***	(1.465,1.601)	1.582***	(1.509,1.658)	1.565***	(1.492,1.641)	1.559***	(1.475,1.627)		1.550***	(1.486,1.638)
White non-Latina Foreign-born	1.781***	(1.726,1.838)	1.829***	(1.769,1.892)	1.739***	(1.681,1.800)	1.765***	(1.704,1.828)		1.709***	(1.650,1.770)
White non-Latina US-born	1.993***	(1.951,2.035)	1.911***	(1.869,1.955)	1.819***	(1.777,1.861)	1.876***	(1.832,1.922)		1.788***	(1.747,1.831)

Even after adjustment, Latina US-born and White non-Latina US-born mothers had 41% (OR=1.407, 95%CI: 1.368, 1.446) and 88% (OR=1.876, 95%CI: 1.832, 1.922) higher odds, respectively, of delivering a macrosomic infant than Black non-Latina US-born mothers. In the unadjusted model (Model 1), White non-Latina foreign-born mothers had 78% higher odds of having a macrosomic baby than Black non-Latina US-born mothers (OR=1.781, 95%CI: 1.726, 1.838), and these odds remained nearly unchanged after adjustment for demographic, medical and socioeconomic risk factors ORs (OR= 1.829, 95%CI: 1.769, 1.892 for Model 2; OR=1.739, 95%CI: 1.681, 1.800 for Model 3). In all models, White non-Latina US-born mothers had higher odds of delivering a macrosomic infant than Black non-Latina US-born mothers (OR=1.993, 95%CI: 1.951, 2.035 for unadjusted Model 1; OR=1.911, 95%CI: 1.869, 1.955 for Model 2; OR=1.819, 95%CI: 1.777, 1.861 for Model 3; OR=1.876, 95%CI: 1.832, 1.922 for Model 4; OR=1.788, 95%CI: 1.747,1.831 (Model 5) (Table 7). Notably, maternal smoking status was adjusted for in Model 5 as smoking status has been previously established to have an association with an increased risk of delivering LBW infants, though multiple studies have demonstrated a non-significant role of maternal smoking on the incidence of macrosomia; this was supported by our findings which showed a negligible impact when adjusting for maternal smoking status between Model 4 and Model 5.

## Discussion

Principal findings

Our data demonstrate a multifactorial association between race, ethnicity, nativity, and macrosomia. We found that White non-Latina US-born mothers had the highest rate of fetal macrosomia and faced significantly higher odds of delivering a macrosomic infant compared to Black non-Latina US-born mothers. US-born mothers of either White or Black race had higher rates of macrosomia than their foreign-born counterparts, even after adjusting for prominent comorbidities and demographic risk factors. This suggests that race, ethnicity, and nativity may represent prognostic factors for fetal size and infant birth weight, independent of the clinical factors previously established to influence macrosomia risk.

Results in the context of what is known

The unique impact of race, ethnicity, or nativity on the prevalence and severity of macrosomia is not well understood. Maternal obesity, gestational weight gain, and diabetes are the most well-established clinical risk factors associated with fetal macrosomia and one might speculate that race-, ethnicity- or nativity-based influences on macrosomia merely predispose to underlying metabolic or diabetic syndromes that lead to birthing LGA infants. Notably, among mothers with significantly elevated BMI, racial or ethnic disparities in macrosomic risk are less evident, however, as BMI trends towards normal pre-pregnancy weight, racial discrepancies in macrosomia risk become more pronounced [22]. The existing research on race or ethnicity and their influence on rates of neonatal birth weights posits a more complicated, independent relationship not solely explained as a predisposition to underlying metabolic disorders. This was emphasized by our previous study, which explored associations between race, nativity, and LBW among Latina and non-Latina mothers in the US and identified maternal race and ethnicity as significant predictors of birth outcomes [20].

Although gestational diabetes and obesity are tightly correlated with an increased risk for macrosomia, our analysis shows that White mothers ultimately had the highest odds of delivering a macrosomic infant despite having lower rates of gestational diabetes or obesity compared with our other cohorts. Our subgroup analysis of macrosomia rates among mothers with pregestational diabetes or gestational diabetes among the racial, ethnic, and nativity cohorts showed that maternal diabetes does not play a mediating role in the relationship between these demographic variables and macrosomia rates. Our findings support those of Ro et al., who reported a cross-sectional study of all live births in the state of New Jersey between 1999 and 2014 (n=1,724,712) [18]. Their research examined birthweight outcomes (i.e., LBW, normal birth weight, and macrosomia) delineated by maternal race and nativity and found that White mothers had the highest proportion of macrosomic infants (11.4%) with no significant difference in macrosomia risk and between foreign- and US-born mothers. Furthermore, White and Asian foreign-born mothers had lower predicted probabilities for macrosomia than White and Asian US-born mothers [18]. Various prospective cohort studies have identified both a greater risk of LBW infants and a decreased risk of macrosomia among Black mothers with a concomitant increased risk of macrosomia among Caucasian mothers [22-26].

Our findings on the relationship between ethnicity and macrosomia were similarly striking. Ethnicity is known to influence BMI and gestational weight gain and thus neonatal birth weights; there is some evidence to suggest that maternal ethnicity impacts infant birth weight in a manner that cannot solely be explained by differences in maternal weight or underlying health conditions [27,28]. In our study, White non-Latina mothers were shown to have notably higher rates of macrosomia compared to Latina mothers or Black non-Latina mothers. Other retrospective cohort studies have found the highest rates of macrosomia among White mothers and higher rates of LGA infants among Latina mothers when compared with Black or Asian mothers, reflecting similar findings in our study [18]. The available literature ultimately displays a more nuanced dynamic between race, ethnicity, and fetal birth weight, which accents the previously-established impact of socioeconomic factors, visceral adipose tissue stores, adaptations to metabolic changes, length of interpregnancy intervals, and maternal age at delivery [22,29,30].

There are numerous possible explanations for why race and ethnicity appear to have an independent impact on macrosomia rates. Genetic differences, including single nucleotide polymorphisms (SNPs) associated with birth weight (e.g., LEKR1/CCNL1, ADCy5, LCORL, 5q11.1, CDKAL1, ADRB1, HMGA1) have been implicated in macrosomia risk [31]. These SNPs may be more prevalent among certain racial or ethnic groups and further study is needed to elucidate whether certain SNPs exist at a higher rate in White maternal populations. Uterine and placental morphology, baseline growth hormone levels such as adiponectin and insulin-like growth factor (IGF), and other genetic variations may be disproportionately present in one racial or ethnic group compared to another and may serve as important biomarkers for macrosomia risk between different racial or ethnic maternal cohorts [32,33]. Variables such as maternal pre-pregnancy hypertension, gestational hypertension, poor nutritional status, environmental exposures, and chronic stress have been well elucidated as contributors to LBW status and are more prevalent in Black and Latina communities [26,34]. We speculate that these risk factors may contribute conversely to decreased risk of macrosomia in our Black and Latina cohorts. Yet in this study, we controlled for these pertinent socioeconomic or demographic factors and continued to identify a similar trend of significant differences in the odds of macrosomia among our racial or ethnic cohorts. Socioeconomic or clinical risk factors, not adjusted for in our linear regression models, including maternal diet, may be more prevalent in certain racial and ethnic groups and thereby underlie the sustained difference in macrosomia risk among our cohorts. These risk factors may serve as lead points for future studies in this domain.

The higher odds of macrosomia among US-born mothers compared to foreign-born mothers in our study may be explained by a corresponding finding of a higher prevalence of advanced maternal age and decreased utilization of supplementary nutritional services among the cohort of women born outside the United States. Additionally, foreign-born mothers have been commonly identified as being at increased risk of receiving diminished access to optimal prenatal care after reaching their country of destination - in part due to lack of health insurance, language barriers, lower socioeconomic class, or undocumented or refugee status [35-38]. Reduced prenatal care has been found to contribute to a higher risk of adverse fetal birth weight outcomes - both LBW and macrosomia [37,39-43]. In our study, advanced maternal age and decreased utilization of WIC services among foreign-born mothers compared to US-born mothers provides the initial evidence for why some foreign-born mothers were found to be at higher risk for fetal macrosomia than US-born mothers, particularly in the context of Black mothers: Black foreign-born mothers had significantly elevated risk of macrosomia compared to Black US-born mothers despite overall lower risk in either cohort compared to White US-born mothers. The basis for racial discrepancies among rates of macrosomia between US-born and foreign-born cohorts, however, is not easily explained. Of note, the disparities between macrosomia rates of US-born individuals and immigrants appear to subside with increased duration of residency and higher rates of macrosomia can be seen among subsequent generations descended from immigrant parents or grandparents [44]. This suggests that acculturation, which is the assimilation and gradual adoption of another culture’s beliefs, values, and practices, may lead the children of many immigrants to grow up with American health and behavioral practices that predispose them to a higher likelihood of birthing macrosomic infants [45].

Clinical and research implications

After adjusting for all significant, established socioeconomic and clinical risk factors for developing fetal macrosomia, White non-Latina US-born mothers had the highest odds of delivering a macrosomic infant while Black non-Latina US-born mothers had the lowest risk of delivering a macrosomic infant. These findings highlight independent racial and ethnic influences over macrosomia risk which potentially refute the traditional understanding of macrosomia as a predominantly diabetes- and obesity-driven disease. Our findings have significant implications for the development of gestational surveillance tools as well as targeted public health interventions, protocols, or policies to improve pregnancy outcomes among particularly high-risk racial, ethnic, and nativity cohorts. Based on the findings of our study, these particularly high-risk cohorts should be more frequently assessed through prenatal ultrasounds, employing sonographic measurements of fetal head circumference [46], abdominal circumference (AC), femur length (FL), and HC/AC ratio to discern the risk of delivering a macrosomic infant. Subsequently, targeted morbidity- and mortality-reducing interventions (e.g., planned labor induction, vaginal delivery at a tertiary hospital with experienced obstetricians, or an elective cesarean delivery) can be discussed and utilized as clinically appropriate.

Further study is required to elucidate the relationship between macrosomia risk and race, ethnicity, and nativity, specifically how these variables influence macrosomia risk both with and without respect to previously established contributing variables such as pre-pregnancy weight, gestational weight gain, and diabetes. Moreover, longitudinal studies are needed to evaluate macrosomia-related sequelae (e.g., childhood or adult obesity, heart disease, and diabetes) among populations of different races or ethnicity. Furthermore, intergenerational studies should be initiated to evaluate the risk for macrosomia among subsequent generations of these different cohorts. 

Strengths and limitations

The predominant strength of our study lies in its utilization of a large dataset of maternal demographics and fetal characteristics associated with 1,791,718 births. This robust dataset allows for multi-variable analysis of fetal macrosomia, providing a means to assess the correlation of large fetal birth weight with race and ethnicity as well as the novel variable of maternal nativity.

The limitations of our current study are similar to the limitations in our previous study investigating the influence of race, ethnicity, and nativity on LBW outcomes [20]. Primarily, our study assessed mothers with self-identified race, which excluded a relatively large number of individuals with ‘presumed’ White or Black race. Numerous racial groups were excluded from this study, which limits the larger conclusions that can be drawn about how other races influence macrosomia risk. In addition, as identified in our previous study, the term ‘Latina’ encompasses a vast array of people from a broad range of nations, cultures, and racial backgrounds; the categorization of these individuals under one ethnic group may impede a more nuanced understanding of how ethnicity influences rates of macrosomia among these otherwise heterogeneous groups. Moreover, Latina individuals in this study were not further stratified by race (e.g., White Latina or Black Latina) and which may limit intervariable comparisons. Latina individuals may also disproportionately self-identify their race as White or not self-identify as any race after listing their ethnicity; this potential quandary in how some individuals self-identify race or ethnicity may have contributed to selection bias which we sought to mitigate through the mutually exclusive groups that were delineated in this study. Future studies should expand these efforts to reduce potential sources of self-identification bias and should aim to include additional racial or ethnic cohorts (e.g., Asian/Pacific Islander, Indian/Alaskan Native) to examine additional trends in macrosomia rates not described in our current study. Socioeconomic variables such as per capita income or social class in the country of birth among foreign-born mothers were not assessed, nor were mothers’ intrauterine environments or the context of any mother’s lifelong experiences (acculturation) leading up to labor and delivery. Although speculative, these missing variables could indirectly play a mediating role in eventual macrosomic birthing outcomes. Maternal diet may be an important contributor to macrosomia risk and we did not have data available to comprehensively impact the potential differences in maternal diet among our cohorts, however, we did examine trends in our data on maternal utilization of WIC services. Lastly, this study on macrosomia rates in the US does not include demographic information from 14 states for which there were insufficient data.

## Conclusions

Our study investigated the association between race, ethnicity, and nativity as independent risk factors for macrosomia in US maternal populations. Our major findings revealed that these factors significantly contribute to macrosomia risk, with White non-Latina US-born mothers experiencing the highest odds of delivering a macrosomic infant. This research adds valuable insight to the limited existing literature on this topic, as national and global rates of macrosomia continue to rise.
